# Laparoscopic combined transperitoneal pelvic exenteration for vulvovaginal recurrence of rectal carcinoma following a Miles operation

**DOI:** 10.1007/s10151-021-02562-0

**Published:** 2022-01-01

**Authors:** H. Zhang, J. Tang, Z. Wei, D. Wang, R. Wang, L. Xiao

**Affiliations:** 1 Department of Gynecology, The First Affiliated Hospital of Chongqing Medical University, Chongqing, 400016 China; 2 Department of Gastrointestinal Surgery, The First Affiliated Hospital of Chongqing Medical University, Chongqing, China; 3 Department of Urological Surgery, The First Affiliated Hospital of Chongqing Medical University, Chongqing, China

**Keywords:** Pelvic exenteration, Recurrent rectal carcinoma, Pelvic mass

Pelvic exenteration refers to a multivisceral resection of all pelvic organs, including the rectum, bladder, and reproductive organs [1]. Approximately 70% of appropriately selected patients can receive R0 resection [2] with a potential of improved quality of life, prolonged survival, and even clinical cure. The attached video shows laparoscopic combined transperitoneal pelvic exenteration for vulvovaginal recurrence of rectal carcinoma following a Miles operation.

The patient is a 37-year-old woman who 5 years previously had undergone a Miles operation for pT1N0M0 stage I rectal adenocarcinoma and, 2 years later was diagnosed with a subcapsular tumor in segment VI of the liver and a pelvic mass involving the right pelvic wall. After three surgeries for metastatic tumors, a hemorrhagic neoplasm in the vulvovaginal region was found on physical examination. Biopsy confirmed locally recurrent rectal adenocarcinoma. The right ureter was involved and a mild hydronephrosis was present. Therefore, we performed a laparoscopic combined transperitoneal pelvic exenteration and bilateral cutaneous ureterostomy in March 2021. The pelvic floor defect was repaired with a biological graft and adjacent soft tissue.

Operation time was 480 min. Blood loss was 600 ml. Complete tumor clearance was achieved without any complications. After multidisciplinary team discussion, adjuvant radiotherapy was suggested as complementary treatment considering the lesion was close to the pubic symphysis and pelvic sidewall. Two months after the surgery, a follow-up computed tomography scan revealed no sign of recurrence or residual lesion. The patient’s quality of life has greatly improved.

Laparoscopic combined transperitoneal pelvic exenteration is technically feasible for a laterally recurrent pelvic tumor and urethral-vaginal fistula after Miles operation and concurrent chemoradiotherapy.

## Supplementary Information

Below is the link to the electronic supplementary material.

Supplementary file1 (MP4 272129 KB)
